# Clinical outcomes of non‐nasopharyngeal lymphoepithelial carcinoma treated with a combined modality approach: A single‐institution study

**DOI:** 10.1002/cam4.5509

**Published:** 2022-12-04

**Authors:** Zichen Qiu, Feifei Lin, Shaowen Lyu, Dehuan Xie, Lei Wang, Zheng Wu, Wanqin Cheng, Yalan Tao, Yong Su

**Affiliations:** ^1^ Department of Radiation Oncology Sun Yat‐sen University Cancer Center, State Key Laboratory of Oncology in South China, Collaborative Innovation Center for Cancer Medicine, Guangdong Key Laboratory of Nasopharyngeal Carcinoma Diagnosis and Therapy Guangzhou People's Republic of China; ^2^ Department of Radiation Oncology (MAASTRO), GROW School for Oncology and Developmental Biology Maastricht University Medical Center Maastricht The Netherlands; ^3^ Department of Radiation Nasopharyngeal Carcinoma Sun Yat‐sen University Cancer Center, State Key Laboratory of Oncology in South China, Collaborative Innovation Center for Cancer Medicine, Guangdong Key Laboratory of Nasopharyngeal Carcinoma Diagnosis and Therapy Guangzhou People's Republic of China; ^4^ Department of VIP Region Sun Yat‐sen University Cancer Center, State Key Laboratory of Oncology in South China, Collaborative Innovation Center for Cancer Medicine, Guangdong Key Laboratory of Nasopharyngeal Carcinoma Diagnosis and Therapy Guangzhou People's Republic of China; ^5^ Department of Radiation Oncology Hunan Cancer Hospital and The Affiliated Cancer Hospital of Xiangya School of Medicine, Central South University Changsha People's Republic of China; ^6^ Department of Radiation Oncology Shunde Hospital of Southern Medical University Foshan P. R. China

**Keywords:** clinical outcome, combined modality, non‐nasopharyngeal lymphoepithelial carcinoma, prognosis

## Abstract

**Background:**

This study presents a summary of the clinical characteristics of non‐nasopharyngeal lymphoepithelial carcinoma (NNPLEC), effects of combined modality treatment and prognostic value of plasma Epstein–Barr virus (EBV) deoxyribonucleic acid (DNA) load, with the aim of providing a reference framework for optimizing treatment practices and outcomes.

**Methods:**

Patients with NNPLEC treated by our center between January 2000 and December 2020 were retrospectively reviewed.

**Results:**

In total, 728 patients were included. The lung was identified as the most common primary tumor site (64.0%), followed by the salivary gland (19.2%). A total of 539 (74.0%) patients underwent surgery, 459 (63.0%) received chemotherapy, and 361 (49.6%) were subjected to radiotherapy. The median follow‐up time was 45 months (range, 6–212 months) and 5‐year overall survival (OS) was 79.1%. Increased plasma EBV‐DNA load of >513.5 copies/mL was predictive of disease progression, with a specificity of 98.1% and a sensitivity of 98.9%. In multivariate Cox analysis, N stage, surgery, and radiotherapy were independent prognostic factors for both OS and PFS. Radiotherapy significantly improves OS in comparison with no radiotherapy group for salivary LEC, while surgery significantly improves OS for pulmonary LEC.

**Conclusion:**

Based on our analysis, surgery and radiotherapy are associated with better OS and PFS for NNPLEC. Radiotherapy could be recommended for salivary LEC, while surgery remains the primary treatment strategy for pulmonary LEC patients. An increased plasma EBV‐DNA load of >513.5 copies/mL is strongly predictive of disease progression, supporting the importance of regular evaluation of plasma EBV‐DNA as part of the diagnostic routine.

## 1 BACKGROUND

Lymphoepithelial carcinoma (LEC), also known as lymphoepithelioma‐like carcinoma, is a poorly differentiated epithelial malignancy featuring tumor‐infiltrating lymphocytes.^1^ LEC often occurs in the nasopharynx (designated undifferentiated nasopharyngeal carcinoma, NPC) with a high incidence in East and Southeast Asia.^2^, ^3^ NPC is sensitive to both radiotherapy and chemotherapy, and current guidelines from the National Comprehensive Cancer Network (NCCN) recommend radiotherapy as the primary treatment modality.^4^ On the other hand, non‐nasopharyngeal lymphoepithelial carcinoma (NNPLEC) is relatively rare and the available literature is mainly based on case reports and studies involving modest sample sizes. To date, no standard guidelines have been developed for the diagnosis and treatment of NNPLEC, and previous studies have generally adopted treatments for other tumors in the same system.^5^, ^6^, ^7^ Detection of plasma Epstein–Barr virus (EBV) deoxyribonucleic acid (DNA) is commonly utilized in clinical practice for the diagnosis of nasopharyngeal carcinoma.^8^, ^9^, ^10^ However, limited research has focused on the prognostic value of plasma EBV‐DNA loads in NNPLEC due to the relative rarity of this disease. To address this gap in knowledge, we have summarized the clinical characteristics of NNPLEC, analyzed the effects of combined modality treatments and examined the prognostic value of plasma EBV‐DNA loads in the present retrospective study, with the aim of providing a reference framework to optimize treatment practices and outcomes.

## 2 MATERIALS AND METHODS

### 2.1 Study population

This study was reviewed and approved by the ethics committee of Sun Yat‐sen University Cancer Center. Patients diagnosed with NNPLEC and treated at our institution between January 2000 and December 2020 were retrospectively reviewed. Cases that met the following criteria were included: (a) gross specimens were from non‐nasopharyngeal tissue and pathologically confirmed as LEC, (b) location of the primary site as well as the exclusion of nasopharyngeal carcinoma were confirmed via imaging and nasopharyngoscopy, followed by nasopharyngeal biopsy or multi‐disciplinary team discussion if necessary, and (c) patients were restaged according to the 8th Edition of the American Joint Committee on Cancer staging system. The exclusion criteria were as follows: (a) the primary site was unknown or the possibility of NPC could not be ruled out, (b) evidence of distant metastasis before treatment or relapse after initial treatment from other hospitals, and (c) incomplete restaging data or follow‐up time of fewer than 6 months.

### 2.2 Treatments

Treatment options for NNPLEC currently include surgery, radiotherapy, and systemic therapy. The selection of treatment depends on the stage, size, location of the disease, patient status, and comorbidities. The irradiation techniques include two‐dimensional (2D) radiotherapy, 3D conformal radiotherapy, intensity‐modulated radiotherapy, and brachytherapy. For patients with locally advanced disease, systemic therapy is needed. The regimens used for patients in this study included combinations of taxol/pemetrexed/gemcitabine/fluorouracil and platinum. Patients with poor performance status received oral fluorouracil. Few patients received immunologic/targeted therapy mainly including epidermal growth factor receptor (EGFR), vascular endothelial growth factor (VEGF)‐targeted monoclonal antibodies (mAb), or tyrosine kinase inhibitors (TKI) and programmed cell death protein 1 (PD1)‐targeted antibodies.

### 2.3 Follow‐up and endpoints

The endpoints were clinical outcomes, specifically, overall survival (OS) and progression‐free survival (PFS). OS was defined as the time from day 1 of treatment to the date of death or patient censoring (whichever occurred first) and PFS as the time to local/regional/distant relapse, death or patient censoring (whichever occurred first). Patients were evaluated once every 3 months within the first 3 years of follow‐up, every 6 months thereafter for 5 years, and then every 12 months until death.

### 2.4 Statistical analysis

Non‐parametric data between two groups were compared with the Mann–Whitney *U*‐test and among three groups with the Kruskal–Wallis test. The Kaplan–Meier method and log‐rank test were used to analyze and compare survival, respectively. The univariate Cox proportional hazard model was applied to calculate hazard ratios. Factors with *p* < 0.10 in univariate analysis were incorporated into the multivariate Cox proportional hazards model. Statistical evaluation of data was conducted using GraphPad Prism, version 7.0 (GraphPad Software, La Jolla, CA, USA).

## 3 RESULTS

### 3.1 Patient characteristics

A total of 728 patients with NNPLEC (362 females (49.7%) and 366 males (50.3%)) were included for analysis of clinical profiles. The age of onset ranged from 11 to 92 years and the median age was 51 years. Around 75% of the patients were non‐smokers. The primary tumor sites were the lung in 465 (63.9%) patients, the salivary gland in 139 (19.0%) patients, and other locations in 0.1%–3.7% of patients. A total of 539 (74.0%) patients received surgery, 459 (63.0%) received chemotherapy, and 361 (49.6%) were administered radiotherapy. Details of patient characteristics are summarized in Table 1.

**TABLE 1:** Characteristics of included patients

Characteristics	Number of cases	Percentage (%)
Total number of patients	728	100
Sex		
Female	362	49.7
Male	366	50.3
Age (Median 51, Range 11–92)		
≤50	355	48.7
>50	373	51.3
Smoke		
Yes	180	24.7
No	548	75.3
T classification		
T1	119	16.3
T2	359	49.3
T3	157	21.6
T4	93	12.8
N classification		
N0	288	39.6
N1	119	16.3
N2	268	36.8
N3	53	7.3
Overall stage		
I	154	21.2
II	143	19.6
III	322	44.2
IV	109	15.0
Surgery		
Yes	539	74.0
No	189	26.0
Radiotherapy		
Yes	361	49.6
No	367	50.4
Chemotherapy		
Yes	459	63.0
No	269	37.0
Targeted therapy		
Yes	46	6.3
No	682	93.7
Immunologic therapy		
Yes	43	5.9
No	685	94.1
Treatment modality		
Surgery alone	223	30.6
Surgery + RT	45	6.2
Surgery + systemic therapy	132	18.1
Surgery + RT + systemic therapy	139	19.1
RT + systemic therapy	176	24.2
Systemic therapy alone or RT alone	13	1.8
Primary sites		
Eye and Orbit	17	2.3
Ear	4	0.5
Nasal cavity and Paranasal sinuses	17	2.3
Salivary gland	138	19.2
Oral cavity	12	1.6
Oropharynx	25	3.4
Hypopharynx	1	0.1
Larynx	1	0.1
Thyroid	3	0.4
Lung	465	64.0
Esophagus	2	0.3
Thymus	27	3.7
Breast	1	0.1
Stomach	5	0.7
Liver	7	1.0
Uterine cervix	1	0.1
Endometrium	1	0.1
Ovarium	1	0.1

### 3.2 Epstein–Barr virus and Human Papilloma virus status

**FIGURE 1:**
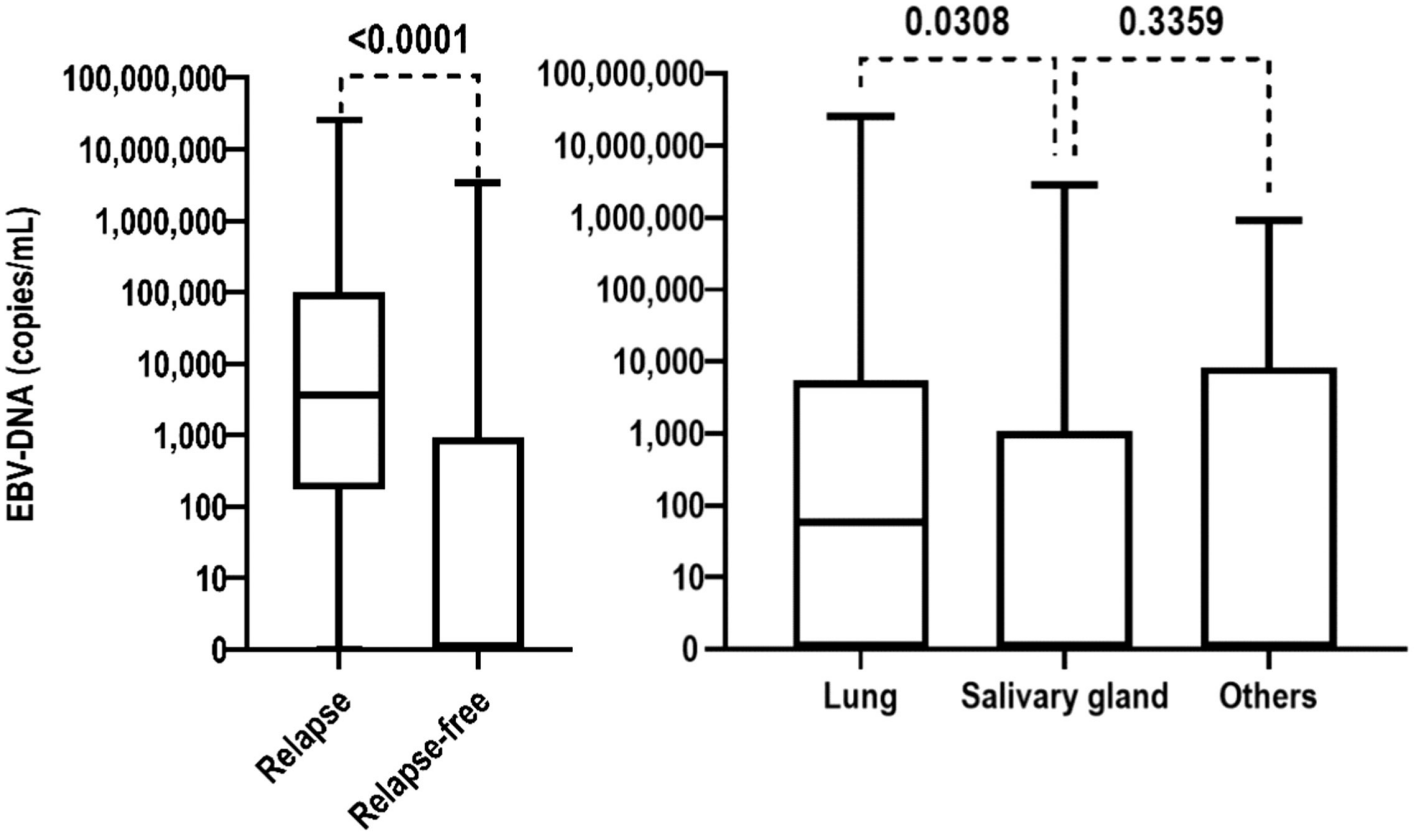
Pre‐treatment EBV‐DNA load.

**FIGURE 2:**
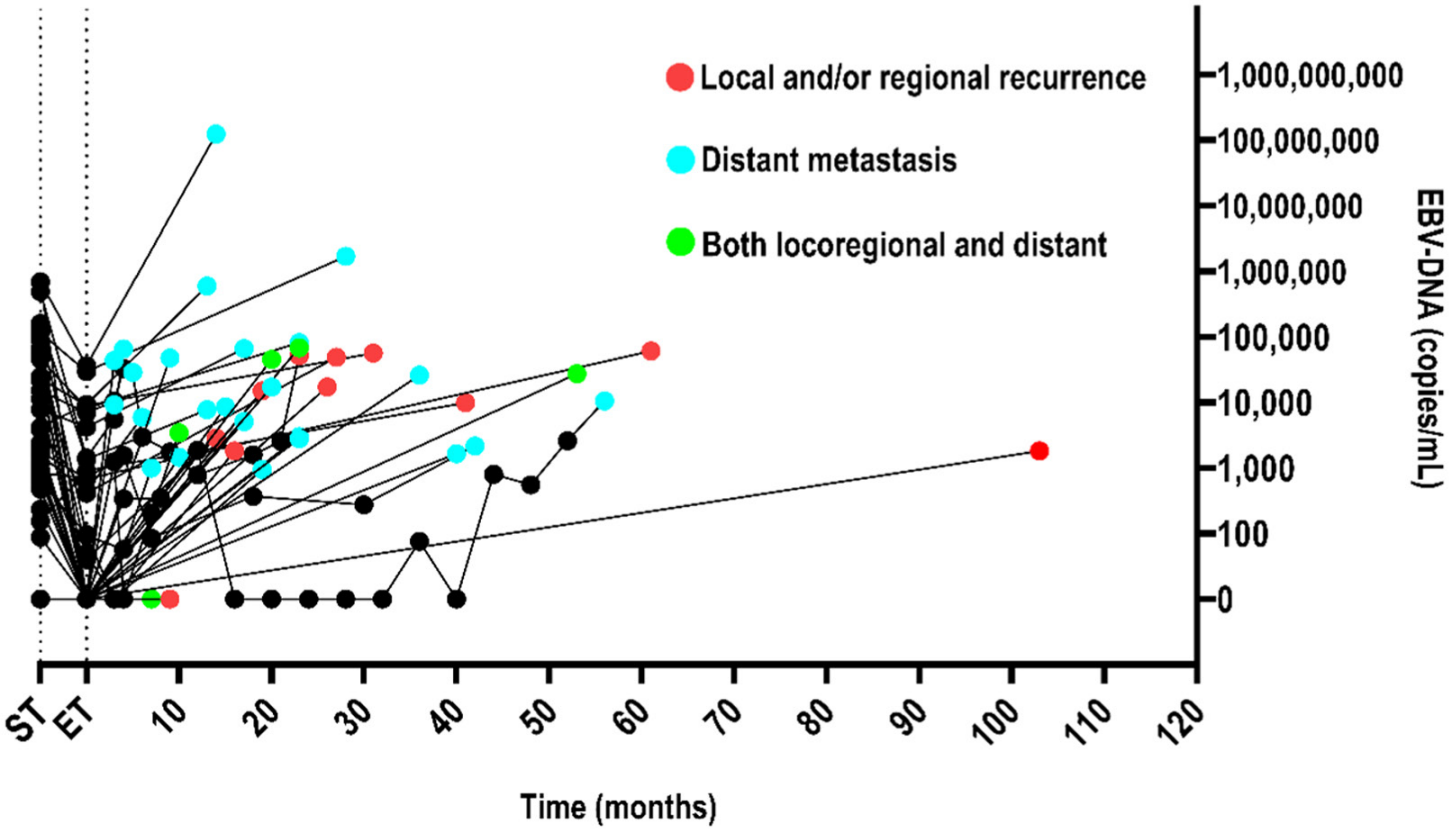
The EBV‐DNA trajectory for the patients with relapses (*N* = 39). The red dot stands for local and/or regional recurrence, and the blue dot stands for distant metastasis. The green dot stands for both locoregional and distant relapses, and the black dot stands for no relapse. ET, end of treatment; ST, start of treatment.

Among the 371 patients presenting plasma EBV‐DNA results before treatment, 195 were tested both before and after treatment and 146 were tested before and after treatment as well as during follow‐up (at least once). Overall, patients displaying relapse had significantly higher plasma EBV‐DNA loads before treatment than those who did not show relapse (median, 3630 vs. 0 copies/mL; *p* < 0.0001). Median pre‐treatment plasma EBV‐DNA loads at different primary sites were estimated as follows: 59 copies/ml in the lung, 0 copies/ml in the salivary gland, and 0 copies/ml in other locations. Furthermore, we observed significant differences in pretreatment plasma EBV‐DNA loads between pulmonary and salivary LEC (Figure 1). The Epstein–Barr virus status of patients is presented in Table 2. Among the 146 patients with plasma EBV‐DNA data before/after treatment and follow‐up, 39 experienced relapses. According to the ROC curve analysis, the cut‐off value of pre‐treatment EBV‐DNA was 2280 copies/ml for OS (sensitivity, 0.655; specificity, 0.798; area under the curve, 0.784; 95% CI of the area under the curve, 0.714–0.854; Figure S1), and the cut‐off value of follow‐up EBV‐DNA was 513.5 copies/ml for OS (sensitivity, 0.989; specificity, 0.981; area under the curve, 0.985; 95% CI of the area under the curve, 0.970–1.000; Figure S1). In cases where the increase in follow‐up plasma EBV‐DNA loads was used to predict relapse. At a cut‐off value of 513.5 copies/ml, specificity and sensitivity for prediction of relapse were 98.1% and 98.9%, respectively. The plasma EBV‐DNA load trajectories for the 39 patients experiencing relapses, including representative plasma EBV‐DNA point data, are illustrated in Figure 2 (point data up to the last follow‐up or confirmation of relapse). Among the six patients (five oropharynx and one cervix) tested for Human Papilloma virus status before treatment, all were negative.

**TABLE 2:** Epstein–Barr Virus status of patients

Variable	Number of cases	Percentage (%)
Pre‐treatment EBV‐DNA (copies/ml)	*N* = 371	100
≤2280	270	72.8
>2280	101	27.2
Relapse during follow‐up	*N* = 371	100
No	291	78.4
Yes	80	21.6
Primary sites	*N* = 371	100
Lung	211	56.9
Salivary gland	88	23.7
Others	72	19.4
Post‐treatment EBV‐DNA (copy/ml)	*N* = 195	100
0	145	74.4
>0	50	25.6
EBV‐DNA during follow‐up	*N* = 146	100
Increasing and >513.5 copies/ml	39	26.7
Not increasing or ≤513.5 copies/ml	107	73.3

### 3.3 Gene status

Among the 456 patients with pulmonary LEC, molecular testing for EGFR was performed in 189 patients and anaplastic lymphoma kinase (ALK) in 160 patients. EGFR mutations or ALK gene rearrangements were only identified in three of the patient samples. One of these patients received gefitinib but experienced progression after 2 months. The gene status details are presented in Table S1.

### 3.4 Clinical outcomes

**FIGURE 3:**
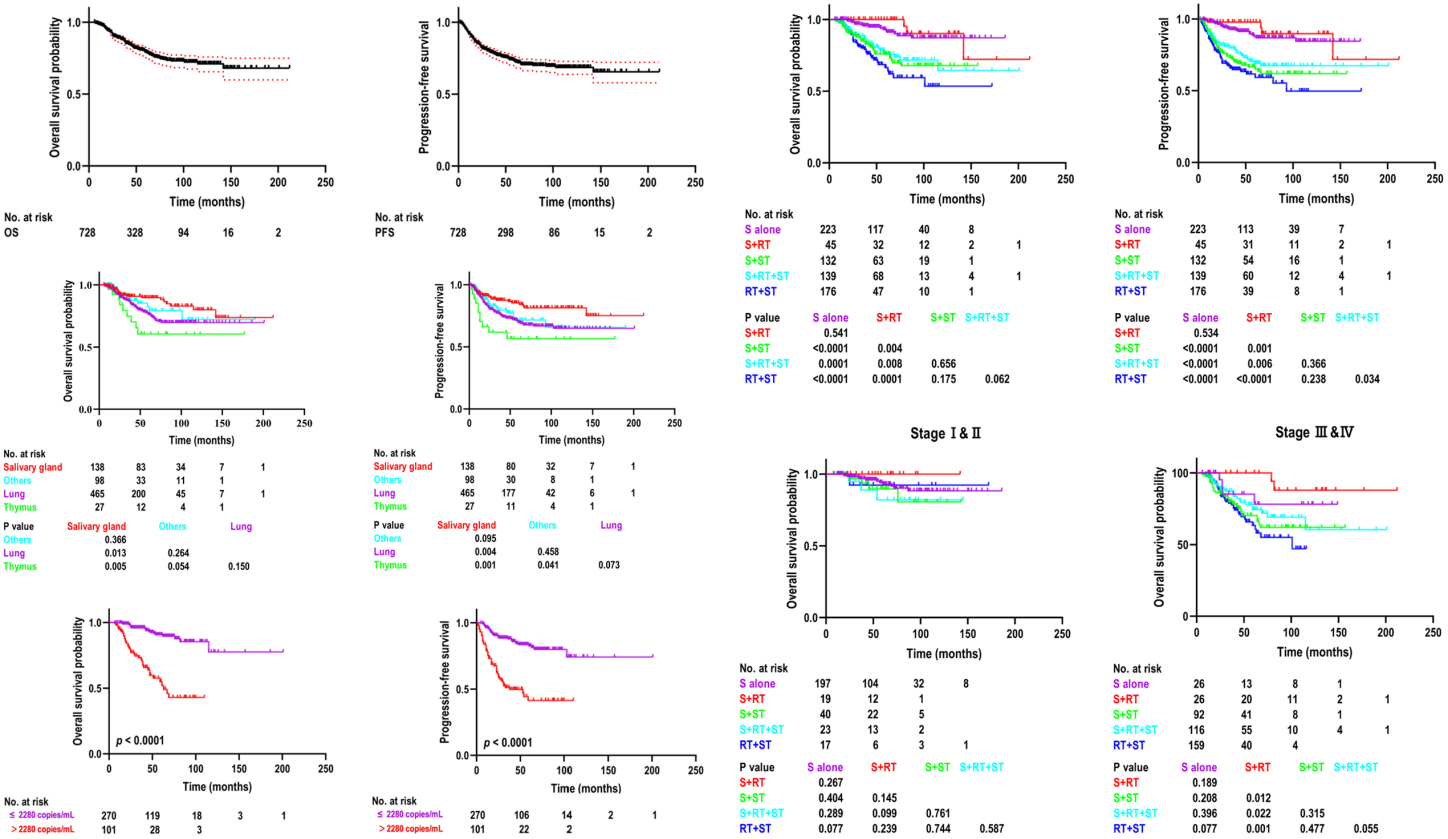
Kaplan–Meier estimates of overall survival and progression‐free survival. Abbreviations: OS, overall survival; PFS, progression‐free survival; RT, radiotherapy; S, surgery; ST, systemic therapy.

The median follow‐up time for NNPLEC patients was 45 months (range, 6–212 months). The last follow‐up date was November 30, 2021, with a loss of follow‐up in 38 patients. Among patients with disease progression (*n* = 157), 57 (36.3%) showed local recurrence only and 85 (54.1%) showed distant metastases only, while both types were detected in 15 (9.6%) patients. The 5‐year OS and PFS rates were 79.1% (95% CI, 75.6–82.6%) and 72.5% (95% CI, 68.8–76.2%), respectively. The corresponding 5‐year OS and PFS rates based on different primary tumor locations were 76.3% and 69.3% for the lung, 89.6% and 85.3% for the salivary gland, 60.4% and 56.7% for the thymus, and 79.3% and 71.7% for other locations. The 5‐year OS and PFS rates were 90.6% and 82.5% among patients with pre‐treatment plasma EBV‐DNA loads of <2280 copies/ml and 54.8% and 41.4% among patients with pre‐treatment plasma EBV‐DNA loads of ≥2280 copies/ml (*p* < 0.0001), respectively. The corresponding 5‐year OS and PFS rates according to different treatment modalities were 92.1% and 86.8% for surgery alone, 100% and 97.8% for surgery combined with radiotherapy, 76.0% and 65.1% for surgery combined with systemic therapy, 75.6% and 69.1% for surgery combined with chemoradiotherapy, and 66.4% and 59.3% for radiotherapy with systemic therapy. The 5‐year OS of patients with stage I/II diseases according to different treatment modalities were 92.9% for surgery alone, 100% for surgery combined with radiotherapy, 89.6% for surgery combined with systemic therapy, 82.0% for surgery combined with chemoradiotherapy, and 92.3% for radiotherapy with systemic therapy. The 5‐year OS of patients with stage III/IV diseases according to different treatment modalities were 78.1% for surgery alone, 100% for surgery combined with radiotherapy, 66.2% for surgery combined with systemic therapy, 74.3% for surgery combined with chemoradiotherapy, and 63.3% for radiotherapy with systemic therapy (Figure 3). The 5‐year OS of patients with pulmonary LEC according to different treatment modalities were 93.7% for surgery alone, 77.3% for surgery combined with systemic therapy, 66.2% for surgery combined with chemoradiotherapy, and 57.4% for radiotherapy with systemic therapy. The 5‐year OS of patients with salivary LEC according to different treatment modalities were 80.1% for surgery alone, 100% for surgery combined with radiotherapy, 85.1% for surgery combined with chemoradiotherapy, and 94.7% for radiotherapy with systemic therapy (Figure S2).

### 3.5 Univariate and multivariate analyses

Univariate analysis showed that female, high T stage, high N stage, high overall stage, radiotherapy, and systemic therapy were associated with the worst 5‐year OS and PFS. Compared with the salivary gland, other primary sites had less favorable 5‐year OS and PFS. Multivariate analysis revealed that N stage, surgery, and radiotherapy were independent prognostic factors for OS and PFS (*p* < 0.05). The results of univariate and multivariate analyses of prognostic factors for OS and PFS are summarized in Table 3. To explore the optimal local treatment strategy for pulmonary LEC and salivary LEC (the most common primary sites), we performed a sub‐group analysis of the primary tumor sites to assess the impact of different treatment modalities on the clinical outcomes. For the patients with pulmonary LEC, surgery significantly improved OS when compared to no surgery patients (HR 0.42, 95% CI 0.23–0.77, *p* = 0.005). For the salivary LEC patients, radiotherapy significantly improved OS in comparison with the no radiotherapy group (HR 0.10, 95% CI 0.01–0.81, *p* = 0.031).

**TABLE 3:** Univariable and multivariable Cox regression analyses

Variable	Overall survival						Progression‐free survival					
	Univariable analysis			Multivariable analysis			Univariable analysis			Multivariable analysis		
	HR	95%CI	*p*	HR	95%CI	*p*	HR	95%CI	*p*	HR	95%CI	*p*
Sex												
Male	1			1			1			1		
Female	1.53	1.08–2.17	0.016	1.42	1.00–2.03	0.053	1.37	1.02–1.85	0.039	1.28	0.94–1.74	0.115
Age												
≤50	1						1					
>50	1.10	0.78–1.54	0.600				1.07	0.79–1.43	0.678			
Smoke												
No	1						1					
Yes	0.90	0.60–1.35	0.902				0.76	0.53–1.10	0.150			
T stage												
T1‐2	1			1			1			1		
T3‐4	2.12	1.50–2.98	<0.0001	1.43	0.96–2.12	0.079	2.01	1.49–2.71	<0.0001	1.23	0.87–1.73	0.237
N stage												
N0‐1	1			1			1			1		
N2‐3	4.35	2.97–6.37	<0.0001	2.50	1.38–4.54	0.003	3.81	2.76–5.27	<0.0001	1.91	1.18–3.10	0.009
Overall Stage												
I / II	1			1			1			1		
III / IV	4.32	2.71–6.90	<0.0001	1.41	0.65–3.06	0.383	4.21	2.82–6.27	<0.0001	1.78	0.93–3.40	0.080
Surgery												
No	1			1			1			1		
Yes	0.34	0.24–0.49	<0.0001	0.53	0.33–0.85	0.009	0.38	0.28–0.51	<0.0001	0.59	0.39–0.89	0.012
Radiotherapy												
No	1			1			1			1		
Yes	1.47	1.04–2.07	0.029	0.59	0.36–0.94	0.028	1.51	1.12–2.04	<0.0001	0.65	0.43–0.98	0.038
Systemic therapy												
No	1			1			1			1		
Yes	4.14	2.57–6.66	<0.0001	1.67	0.89–3.16	0.114	4.05	2.68–6.11	<0.0001	1.72	0.99–2.99	0.052
Primary site			0.022			0.635			0.006			0.366
Salivary gland	1			1			1			1		
Others	1.34	0.66–2.74	0.423	0.59	0.25–1.36	0.215	1.61	0.88–2.93	0.121	0.51	0.24–1.10	0.085
Lung	1.88	1.13–3.12	0.015	0.63	0.27–1.48	0.287	1.92	1.21–3.02	0.005	0.67	0.32–1.40	0.283
Thymus	3.03	1.36–6.74	0.007	0.71	0.34–1.46	0.347	3.33	1.61–6.86	0.001	0.72	0.38–1.38	0.324

## 4 DISCUSSION

LEC of non‐nasopharyngeal tissues is an exceedingly rare malignancy. Here, we reviewed the clinical characteristics of NNPLEC, analyzed the effects of combined modality treatments and determined the prognostic value of plasma EBV‐DNA load with the aim of providing a reference framework to optimize treatment practices and outcomes. To our knowledge, the current study includes the largest NNPLEC sample size from a single center.

While radiotherapy has emerged as the primary treatment modality for patients with nasopharyngeal LEC, no consensus has been reached on therapy for NNPLEC. A retrospective cohort study using the Surveillance, Epidemiology, and End Results (SEER) database analyzed 1,184 patients with nasopharyngeal LEC and 922 patients with NNPLEC, which concluded that radiotherapy could be recommended for nasopharyngeal LEC due to its significant survival benefit while surgery should remain the primary treatment strategy for resectable non‐nasopharyngeal LEC.^11^ A number of studies targeting specific locations of LEC (such as lung, salivary gland, cervix, and larynx/hypopharynx) have reported similar results, advocating surgery as the primary modality.^7^, ^12^, ^13^, ^14^, ^15^ However, data from our study identified both surgery and radiotherapy as independent prognostic factors for OS and PFS. And the sub‐group analysis of treatment modalities on outcomes of pulmonary and salivary LEC showed that radiotherapy significantly improves OS in comparison with the no radiotherapy group for salivary LEC, while surgery significantly improves OS for pulmonary LEC. In view of the available treatments for NPC, we speculated whether NNPLEC could benefit from radiotherapy. A number of previous studies indicate that NNPLEC is sensitive to radiotherapy. A report by Lin and co‐workers on 25 patients with advanced pulmonary LEC who received radiotherapy after first‐line chemotherapy revealed significantly longer PFS and OS relative to patients not subjected to radiotherapy.^16^ A study on 34 patients with NNPLEC of the head‐and‐neck by Praveen et al.^2^ further supported the utility of radiotherapy as appropriate initial locoregional therapy. Definitive chemoradiotherapy also achieved good results in cases of NNPLEC of the nasal cavity, middle ear, salivary gland, oropharynx, larynx/hypopharynx, and vagina.^16^, ^17^, ^18^, ^19^, ^20^, ^21^ Comparable results to the present study were obtained by another retrospective cohort study on the SEER database involving 378 patients with head‐and‐neck NNPLEC. Radiation was identified as an independent prognostic factor for both OS and disease‐specific survival.^22^ In view of these collective results, we hypothesize that NNPLEC may benefit from radiotherapy similar to NPC. Also based on our current results, radiotherapy could be recommended for salivary LEC, while surgery remains the primary treatment strategy for pulmonary LEC patients. Further prospective studies are warranted to provide stronger evidence and validate the current findings.

Studies to date have confirmed that chemotherapy confers survival benefits for NPC patients. Guidelines from the NCCN recommend chemoradiotherapy combined with induction chemotherapy or adjuvant chemotherapy as the primary treatment for advanced NPC.^4^ Further reports have confirmed that chemotherapy is also effective for pulmonary LEC.^15^ The majority of non‐chemotherapy cases included in the present study were early stage with good prognosis, and therefore, the cohort failed to reflect the survival benefits of chemotherapy. Thus, the efficacy of chemotherapy for NNPLEC and potential optimal regimens require further investigation. In our experiments, univariate analysis showed an association of the primary site with prognosis while no significant differences were evident with multivariate analysis, which could be attributable to several potential factors. On the one hand, the variable number of cases involving different primary sites may lead to biased results. On the other hand, the results suggest that regardless of the primary site, LEC should be treated with combined modality treatments to improve prognosis owing to its presentation as a poorly differentiated malignancy with strong local invasion and metastasis tendency.

Recently, targeted therapy has been gradually applied to NPC, but experience in application to NNPLEC is limited because of the rarity of the disease. In a retrospective study by Wang et al.^23^ a regimen containing nimotuzumab improved the long‐term survival of patients with stages III‐IV NPC. Xue and co‐workers conducted a phase II prospective clinical trial in patients with recurrent or metastatic NPC. Their results showed that OS with the regimen containing sorafenib was not improved compared with previous studies but good results were obtained in patients with lung metastasis (median OS, 20.9 vs. 11.7 months, *p* = 0.05).^24^ Another phase II study by Chua et al.^25^ reported that patients with recurrent or metastatic NPC had a poor response rate to gefitinib. While targeted therapy has beneficial prospects in LEC, accurate screening of beneficiaries remains an urgent problem in clinical practice. Targeted therapy is closely related to driver genes, which are widely reported in lung cancer. Therefore, the determination of the gene status of pulmonary LEC may provide a reference framework for the treatment of LEC at other primary sites. Yin et al.^26^ conducted a study on 330 patients with pulmonary LEC with an EGFR mutation rate of 2.9% (5/175) and ALK alteration rate of 2.1% (3/140) and retrospectively analyzed 1071 pulmonary LEC cases from a total of 15 articles identified from electronic searches, among which 15 contained EGFR mutations, including four patients who showed rapid progression after receiving EGFR‐TKI. The present study additionally revealed very low mutation rates of common driver genes in pulmonary LEC and almost no response of mutation‐positive cases to EGFR‐TKI. The collective evidence suggests an unsatisfactory response of NPC and pulmonary LEC to EGFR‐TKI. Data from whole‐exome sequencing, targeted deep sequencing, and single‐nucleotide polymorphism arrays by Hong and co‐workers revealed that pulmonary LEC resembles NPC more closely relative to other lung cancers,^27^ which may explain the similar responses of lung LEC and NPC to EGFR‐TKI.

Immunotherapy as a potential treatment option for LEC is emerging as a research hotspot. An earlier phase I single‐arm clinical trial in patients with recurrent or metastatic NPC showed overall response rates of patient groups treated with PD‐1 mAb alone and combined with GP of 34% and 91%, respectively.^28^ Other studies have reported PD‐L1‐positive rates in patients with pulmonary LEC of 63.3–75.8% and case reports suggest that pulmonary LEC responds favorably to PD‐1 inhibitors.^29^, ^30^, ^31^, ^32^ Among the patients included in the present study, 43 were treated with PD‐1 inhibitors but none were tested for PD‐L1 expression, leading to difficulty in the analysis of efficacy. Therefore, the utility of immunotherapy in this patient population remains to be established.

The relationship between EBV and LEC has additionally received widespread attention. Considerable evidence supports the crucial role of EBV infection in the pathogenesis of NPC. Lin and co‐workers demonstrated that plasma EBV‐NDA reflects the tumor load of NPC. Patients showing relapse had a significantly higher plasma EBV‐DNA concentration before treatment than those without relapse (median, 3035 vs. 1202 copies per milliliter; *p* = 0.02). OS and relapse‐free survival were significantly lower among patients with pretreatment plasma EBV‐DNA concentrations of ≥1500 copies per milliliter relative to those with <1500 copies per milliliter.^33^ A related report of EBV‐DNA in LEC outside the nasopharynx is additionally documented in the literature. Li and co‐workers confirmed that increased plasma EBV‐DNA >1000 copies/ml is strongly predictive of pulmonary LEC progression within 3 months, with a specificity of 97.5% (95% CI: 86.8%–99.6%), and associated with impaired OS (2‐year OS, >1000 and ≤ 1000 copies/ml, 72.9% and 100%, *p* < 0.001).^34^ In the present study, we further investigated the prognostic value of plasma EBV‐DNA load in NNPLEC. Notably, OS and PFS were significantly lower among patients with pretreatment plasma EBV‐DNA loads of ≥2280 copies/ml relative to those with loads of <2280 copies/ml. Patients showing relapse had significantly higher plasma EBV‐DNA concentrations before treatment than those with no relapse (median, 3630 vs. 0 copies per milliliter; *p* < 0.0001). Increased EBV‐DNA loads exceeding 513.5 copies/ml were strongly predictive of disease progression. Although the detection of plasma EBV‐DNA loads is not a routine clinical procedure at present, our results support the regular testing of plasma EBV‐DNA as part of the prognostic workup for NNPLEC.

The present study has several limitations that should be taken into consideration. First, the rarity of NNPLEC presents an obstacle in terms of collecting large‐scale homogenous data. Comprehensive analysis of patients at relatively different stages of disease progression, diverse treatments, and irradiation techniques is necessary to include all the different primary sites. Second, the detection of plasma EBV‐DNA load is not a routine clinical procedure and missing data could lead to bias. Third, none of the patients treated with PD‐1 inhibitors were tested for PD‐L1 expression.

## 5 CONCLUSION

Based on our analysis, surgery and radiotherapy are associated with better OS and PFS for NNPLEC. Radiotherapy could be recommended for salivary LEC, while surgery remains the primary treatment strategy for pulmonary LEC patients. An increased plasma EBV‐DNA load of >513.5 copies/ml is strongly predictive of disease progression, supporting the importance of regular evaluation of plasma EBV‐DNA as part of the diagnostic routine.

## AUTHOR CONTRIBUTIONS

**Zichen Qiu:** Conceptualization (lead); data curation (lead); formal analysis (lead); investigation (lead); methodology (lead); writing – original draft (lead); writing – review and editing (lead). **Feifei Lin:** Data curation (equal); formal analysis (equal); investigation (equal); methodology (equal). **Shaowen Lyu:** Data curation (supporting); formal analysis (supporting); investigation (supporting); methodology (supporting). **Dehuan Xie:** Data curation (supporting). **Lei Wang:** Data curation (supporting). **Zheng Wu:** Formal analysis (supporting). **Wanqin Cheng:** Investigation (supporting). **Ya‐lan Tao:** Conceptualization (equal); project administration (equal); resources (equal); supervision (equal); writing – review and editing (equal). **Yong Su:** Conceptualization (lead); investigation (lead); project administration (lead); resources (equal); supervision (equal); writing – review and editing (equal).

## FUNDING INFORMATION

The authors declare that they have no funding.

## CONFLICT OF INTEREST

The authors declare that they have no competing interests.

## ETHICS APPROVAL AND CONSENT TO PARTICIPATE

This study was approved by the Medical Ethics Committee of Affiliated Hospital of Sun Yat‐sen University Cancer Center, and the need for written informed consent was waived.

## Supporting information


Figure S1.
Table S1.

## Data Availability

All relevant data were uploaded onto the Research Data Deposit public platform (www.researchdata.org.cn) with approval number of RDDA2022715901.
