# Supplementary material for: Clinical outcomes of non‐nasopharyngeal lymphoepithelial carcinoma treated with a combined modality approach: A single‐institution study
Source: Cancer Med. 2022 Dec 4;12(6):7105–15. doi: 10.1002/cam4.5509 (PMC10067105; doi:10.1002/cam4.5509)
Supplement: Supplementary file 1 — Figure S1. Table S1. [file CAM4-12-7105-s001.docx]

Supplementary Figure 1. ROC curve


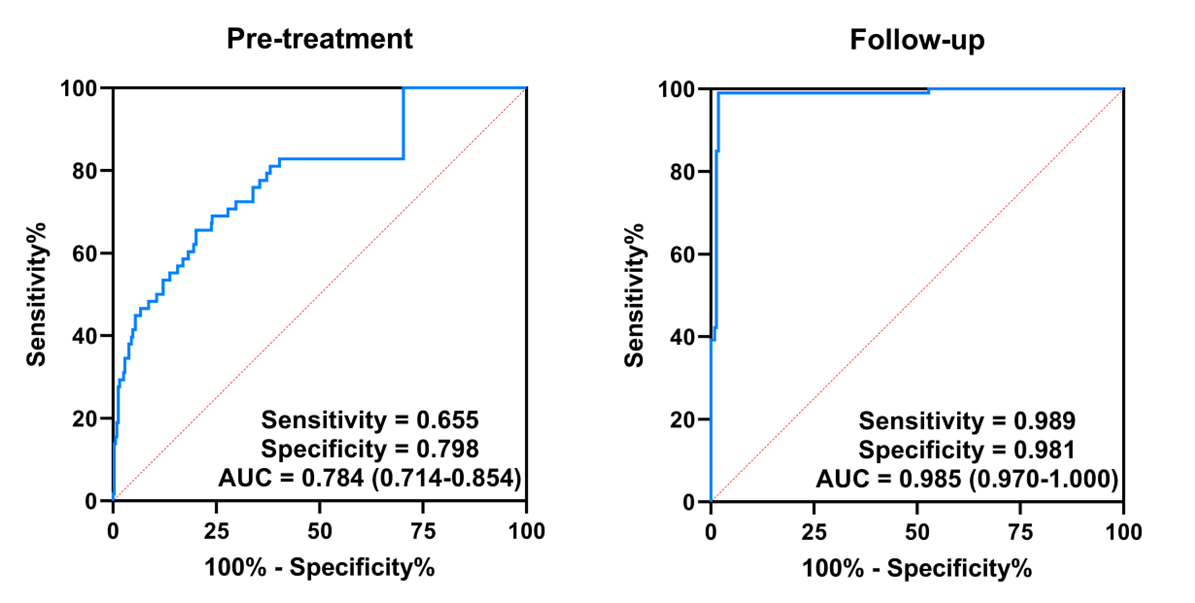


Supplementary Figure 2. Kaplan-Meier estimates of overall survival


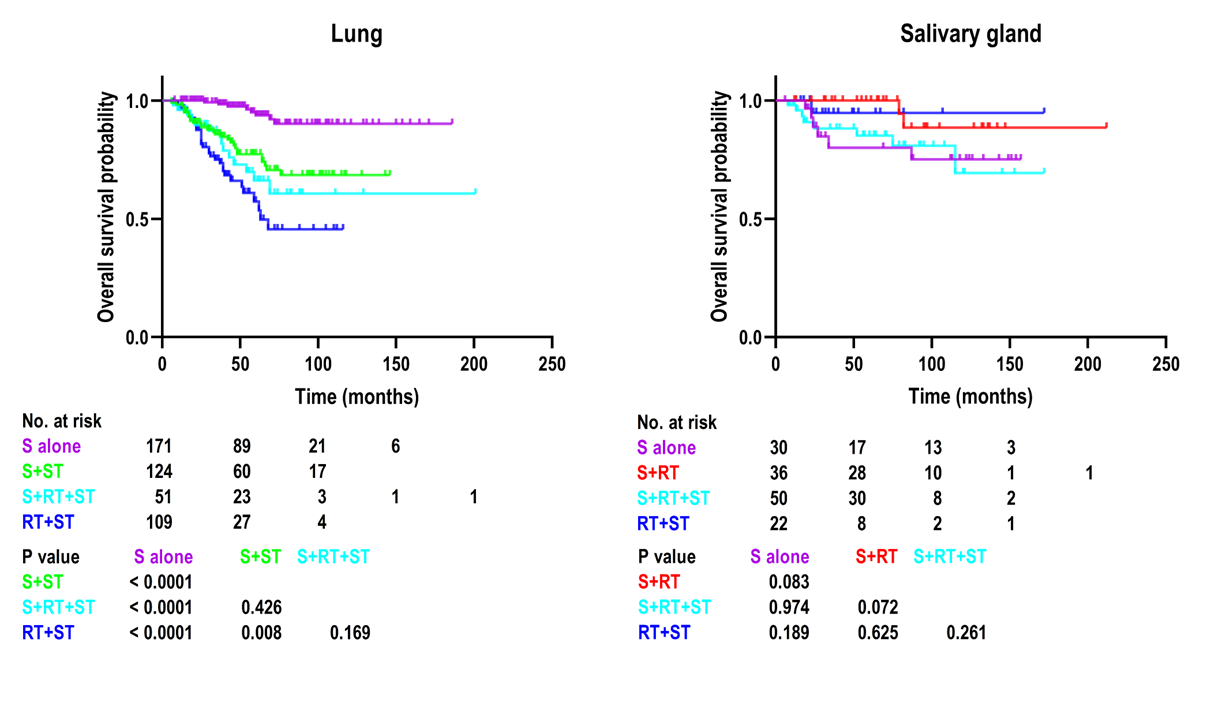


Supplementary Table 1. Gene status of EGFR/ALK-positive patients

| Case | Gene status | Targeted therapy | Time to progression after targeted therapy |
| --- | --- | --- | --- |
| 1 | EGFR 21-L858R | / | / |
| 2 | EGFR 19-E746_A750del | Gefitinib | 2 months |
| 3 | EML4-ALK | / | / |
